# Acquired von Willebrand Syndrome in Patients With Ventricular Assist Device

**DOI:** 10.3389/fmed.2019.00007

**Published:** 2019-02-05

**Authors:** Antoine Rauch, Sophie Susen, Barbara Zieger

**Affiliations:** ^1^INSERM, U1011, Univ. Lille, U1011-EGID, Institut Pasteur de Lille, Lille, France; ^2^CHU Lille, Hematology and Transfusion, Lille, France; ^3^Division of Pediatric Hematology and Oncology, Department of Pediatrics and Adolescent Medicine, Faculty of Medicine, Medical Center-University of Freiburg, University of Freiburg, Freiburg, Germany

**Keywords:** ventricular assist devices, von Willebrand factor, acquired von Willebrand syndrome, ECMO, bleeding

## Abstract

During the last decade the use of ventricular assist devices (VADs) for patients with severe heart failure has increased tremendously. However, flow disturbances, mainly high shear induced by the device is associated with bleeding complications. Shear stress-induced changes in VWF conformation are associated with a loss of high molecular weight multimers (HMW) of VWF and an increased risk of bleeding. This phenomenon and its cause will be elaborated and reviewed in the following.

## Introduction

In 1958, Edward Heyde observed a link between the presence of aortic stenosis (AS) and gastrointestinal (GI)-bleeding of idiopathic origin in 10 patients (1, 2). Since this original description, the association between GI-bleeding due to angiodysplasia and various cardiac defects has been confirmed by several groups.

A major discovery was the identification of a loss of high molecular weight multimers (HMW) of von Willebrand factor (VWF) in patients with congenital and acquired AS (3, 4). Warkentin et al. suggested in 1992 that VWF could be the missing link between AS and the GI-bleeding due to angiodysplasia (5) also implying a causal relation between aortic gradient, and the decrease of HMW-multimers. The defect in VWF multimers is not unique to AS, but is also manifested in several other heart conditions, including hypertrophic cardiomyopathy (6) and aortic or mitral regurgitation (7, 8). Most illustrative is the condition where patients are exposed to continuous flow ventricular assist device (CF-VAD), as a loss of HMW-multimers is observed in all of these patients (9, 10). This phenomenon and its cause will be elaborated and reviewed in the following.

## Von Willebrand Factor

**Figure 1:**
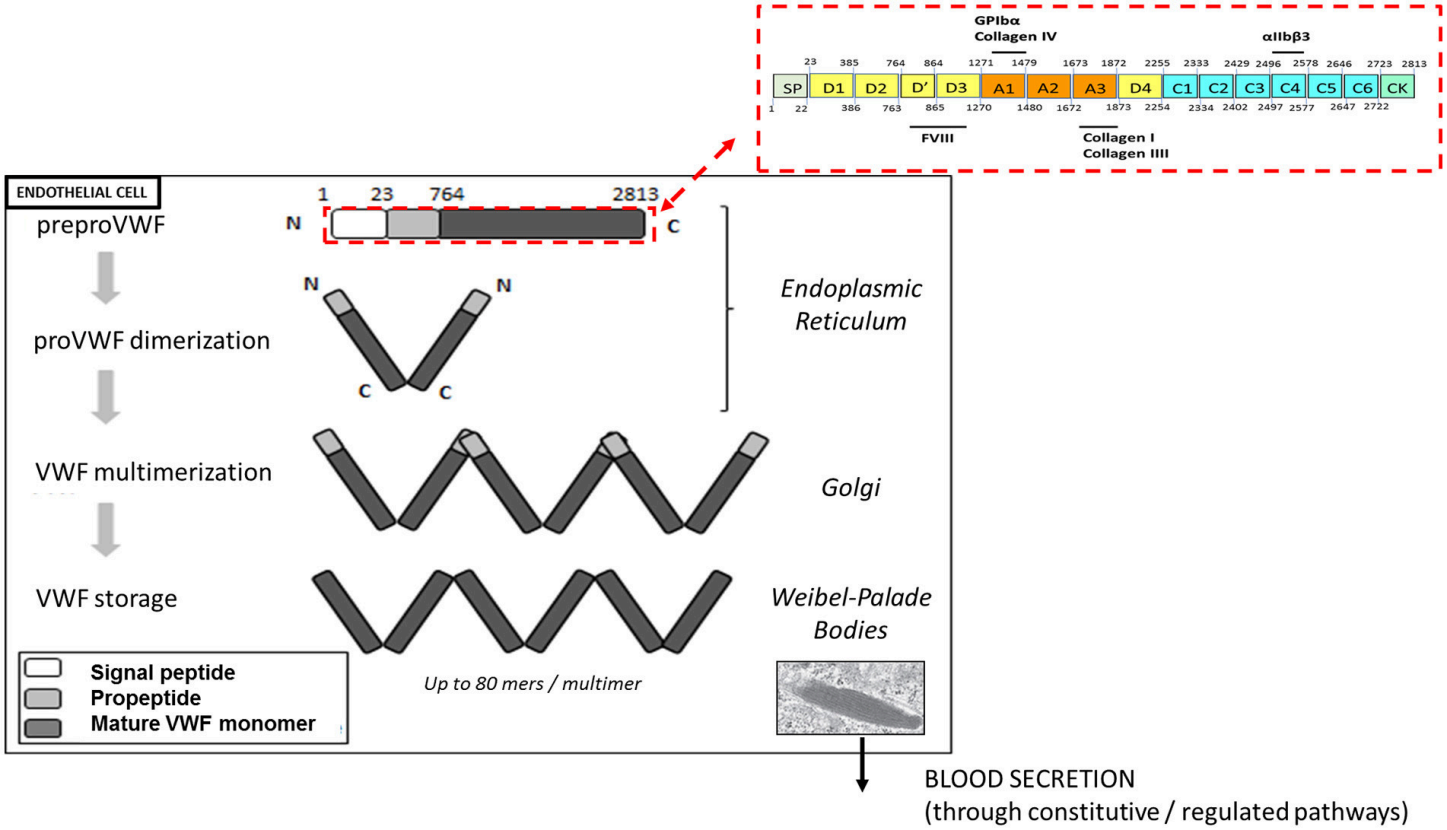
Biosynthesis and storage of VWF multimers in endothelial cells. VWF is synthetized as a prepropolypeptide of 2,813 amino acids with the following updated domain structure: D1-D2-D'-D3-A1-A2-A3-D4-B1-B2-B3-C1-C2-CK. After peptide signal removal, proVWF subunits undergo disulfide bridging through their C-terminal CK domains. In the Golgi apparatus, the acidic pH promote the organization of the prodimers in a conformation favoring VWF multimerization via disulfide bridging of adjacent N-terminal D3 domains. In addition, furin mediates the proteolytic separation of the propeptide from the mature VWF subunits. Following synthesis, VWF multimers are stored in the Weibel-Palade bodies before their basal or regulated secretion from endothelial cells.

VWF plays a major role in primary hemostasis by promoting the adhesion of platelets to subendothelial collagen at sites of vascular damage and thereby promoting platelet aggregation. VWF is a plasma glycoprotein, synthesized by endothelial cells and megakaryocytes in a multimerized form. VWF is transcribed as a single-chain prepropolypetide of 2,813 amino acids, consisting of a signal peptide, a large propeptide, and the mature VWF subunit. The primary sequence of VWF is composed of different structural and functional domains arranged in the order D1-D2-D′-D3-A1-A2-A3-D4-C1-C2-C3-C4-C5-C6-CK, with the D1-D2 domains representing the propeptide and the remainder corresponding to the mature VWF subunit (Figure 1) (11).

After removal of the signal peptide, the VWF units dimerize via their CK-domains and are transported into the Golgi apparatus, where they undergo N-terminal disulfide bonds formation involving cysteine residues within the D3 domain. This multimerization process generates a pool of differentially sized VWF multimers that may be composed of as many as 80 mers (12). In addition, the propeptide is cleaved from mature VWF subunits by furin, but remains non-covalently associated. VWF multimers and propeptide are then transported and packaged into specific storage organelles: the Weibel-Palade bodies (WPBs) in endothelial cells and the α-granules in megakaryocytes /platelets (12).

Each monomer contains multiple binding sites: the A1 domain is the binding site for platelet glycoprotein Ibα (GPIbα) and for collagen IV, the A3 domain is the binding site for collagens I and III and the C4 domain is the binding site for the platelet-integrin αIIbβ3 (Figure 1). Each of these interactions is relevant to support the hemostatic function of VWF in order to stop the bleeding.

VWF multimers are released from platelet α-granules upon platelet activation. Endothelial cells secrete VWF constitutively to maintain basal levels of VWF in plasma or through a regulated pathway from WPBs after endothelial stimulation (12). Much of the secreted VWF remains bound to the endothelial surface until being proteolytically removed by the metalloprotease A Disintegrin-like And Metalloprotease ThromboSpondin-13 (ADAMTS13). Endothelium-attached VWF multimers unfold under fluid shear stress and flow acceleration, thereby becoming more adhesive to bind platelets. Unfolding of the multimers further exposes the cleavage site for ADAMTS13 within the A2 domain. VWF proteolysis by ADAMTS13 results in the presence of smaller multimers VWF in the circulation thus reducing the thrombogenic potential of VWF. Failure to remove endothelium-bound VWF in case of thrombotic thrombocytopenic purpura (TTP) secondary to congenital or acquired ADAMTS13 deficiency allows individual VWF multimers to self-associate to form long strands (up to several hundred μm long) that promote platelet adhesion and thrombus formation leading to microvascular occlusion (13).

Once the VWF A2 domain is unfolded, it can also be cleaved *in vitro* in a shear-dependent manner by other proteases than ADAMTS13, such as thrombin, plasmin (14) or leukocyte proteases (15). However, the *in vivo* relevance of leukocytes proteases in VWF proteolysis is questionable since VWF fragments remain observed after depletion of neutrophils in Adamts13^−/−^ mice or in neutropenic patients (16). Recent studies in mice models reported that plasmin can function as a back-up for ADAMTS-13 to proteolyze VWF and mitigate TTP symptoms, but only in conditions of supraphysiological increased plasmin activity to overcome the inhibition by α2-antiplasmin and plasminogen activator inhibitor 1 (PAI-1) (17).

## Shear and Von Willebrand Factor

Its exceptionally large size makes the VWF multimers sensitive to the influence of biophysical forces. Changes in shear stress forces impose rapid changes in the conformation of the VWF multimers. In fact, shear stress forces play a crucial role in regulating the hemostatic activity of VWF. At increasing shear rates and regions of flow acceleration, VWF gets uncoiled with extended multimers attaining lengths up to ~1,300 nm. Via end-to-end self-association, these uncoiled multimers may form fibrils that can be hundreds of microns long (18).

Shear stress-induced shape changes unveil a cryptic binding site for GPIbα within the A1 domain. The multimeric structure of VWF strongly promotes GPIbα interactions by increasing avidity (19, 20). The conformational changes also induce unfolding of the A2 domain, creating the substrate for ADAMTS13 by exposing the scissile bond Tyr1605-Met1606 to ADAMTS13, which cleaves HMW-multimers into smaller ones that have lesser hemostatic activity (20). *In vitro* studies revealed that unfolding of the A2 domain, which is required for proteolysis, occurs in <1 s. It was further inferred that unfolding and cleavage of the VWF A2 domain *in vivo* could occur within 200 s in response to acute changes in shear conditions (21). This unique mechano-enzymatic regulation results in high shear rate provoking an almost immediate shift in VWF-size distribution to smaller VWF-multimers and subsequent counter-regulation of its hemostatic effect (13). Lack of VWF cleavage resulting from ADAMTS13 defects leads to diffuse microvascular thrombosis promoted by the increased binding of A1 domain of VWF to its platelet-receptor GPIbα (13). Conversely, increased VWF susceptibility for ADAMTS13 is a feature of VWD-type 2A, characterized by a mucosal bleeding phenotype secondary to the loss of HMW-multimers (22).

It should be noted that besides high shear rates, platelet binding increases the sensitivity for ADAMTS13-mediated proteolysis (23). It is not surprising therefore that gain-of-function mutations that promote binding to GPIbα (qualified as VWD-type 2B) are associated with increased proteolysis (24). There is thus a delicate balance between the need of VWF to unfold in order to unveil its A1 domain for platelet binding, and the unfolding of the A2 domain that exposes the ADAMTS13 cleavage site (13).

Shear stress-induced changes in VWF conformation have been shown to occur above the threshold of 1,000 s^−1^ in laminar flow (25), corresponding to shear observed in non-stenotic small arteries and arterioles (26).

## Acquired VWF Defects in Cardiac Devices: *in vitro* Models

CF-VADs and extracorporeal membrane oxygenation (ECMO) are associated with a complete loss of HMW-multimers and in some cases even a decrease in intermediate multimers (10, 27, 28). The initial time course of loss/recovery of HMW-multimers after acute changes in blood flow *in vivo* and the mechanisms associated with these changes are still a matter of debate. Dedicated pre-clinical models have been developed to investigate the initial time course of loss/recovery and to better understand the potential pathways that can be targeted to reduce bleeding complications. These *in vitro* models were translational mechanical circulatory support (MCS) mock circulatory loops perfused with whole blood or platelet-rich plasma. Using data derived from these models, it was initially interpreted that in this setting, VWF degradation was mainly due to the mechanical destruction of VWF when circulating through the assist device, with no or limited role of enzymatic degradation (29). More recent and comprehensive studies performed in other models demonstrated that there was a specific loss of HMW-multimers starting very early after the initiation of the pump. Loss of HMW-multimers seems related to the rotational speed of the device, and is associated with a proteolytic degradation depending on both ADAMTS13 activity and platelets (30, 31). In those models, inhibition of HMW-multimers degradation by an antibody specific to the D4 domain of VWF, interfering with ADAMTS13-mediated cleavage, further underscored the key role of ADAMTS13 proteolysis (32). According to Nascimbene et al. (33), the loss of HMW-multimers in patients on LVAD support was observed in 91.3% in their case series. However, excessive VWF cleavage by ADAMTS-13 was found in only 20% of them. Therefore, they suggested that shear-induced VWF binding to platelets may be an alternative cause of LVAD-associated AVWS. They showed that among 10 normal subjects examined, high shear stress induced the loss of large VWF multimers in 7 without increasing VWF cleavage by ADAMTS-13 (33). HMW-multimers defect under continuous flow VAD might therefore result not only from an increased cleavage by ADAMTS13 (“VWD-type 2A like” mechanism) but also from an increased binding to circulating platelets (“VWD-type 2B-like” mechanism). Whether the latter gain of function mechanism could contribute to thrombotic risk in VAD-patients, through promotion of platelet activation and aggregation, remains to be confirmed.

## Onset and Offset of Acquired VWF Defects in Cardiac Devices *in vivo*

Rapid degradation has also been observed *in vivo*, and early reports describing a calve model implanted with a continuous flow MCS (34) suggested that an increased secretion of ADAMTS13 from endothelial cells near the anastomosis could explain the decrease in HMW-multimers. *In vitro* and *in vivo* studies differ with regard to the recovery of HMW-multimers upon reversal of the high shear situation: no “re-multimerization” of VWF occurs in MCS mock circulatory loops, whereas a rapid recovery of VWF multimers is observed in dedicated animal models (30). For instance, in a rabbit model of reversible aortic stenosis (aortic banding), an acute onset/offset (within 5 min) of the HMW-multimers defect was observed (30). Since no recovery of HMW-multimers occurs when performing whole blood perfusion experiments in the absence of endothelium in the MCS mock circulatory loop model, it seems conceivable that endothelial cells provide the source HMW-multimers. Moreover, the recovery of the pool of HMW-multimers in plasma is too fast to be explained by the basal endothelial secretion. Instead, it seems likely that there is a rapid release of VWF from WPBs endothelial storage-pools. It was investigated in a swine CF-VAD model whether sequential and dynamic changes of vascular arterial luminal pressure in combination with high shear could modulate VWF multimerization over time. This study demonstrated that changes in pulsatility regimen can be pivotal to modulate VWF multimerization in plasma in high shear condition (35). Indeed, acute changes of pulse pressure induced a release of new HMW-multimers from WPBs, as well as other bioactive compounds stored in WPBs, such as factor VIII and angiopoietin-2 (Ang-2) (35). Indeed, other studies have demonstrated that an increase in the arterial luminal pressure is able to induce an acute release of VWF by the vascular endothelium (36).

## VAD Support for Bridging, for Recovery and for Destination Therapy

During the last decade the use of CF-VADs for patients with severe heart failure has increased tremendously. About 15% of the patients who are listed for heart transplantation (HT) decease before the transplant may be available. The number of patients waiting for HT has doubled during the last 15 years. However, the availability of the hearts transplants decreased by a third (37). As a consequence, more than 30% of the patients waiting for HT need a VAD as bridge to HT. Moreover, for patients with end-stage heart failure too old for transplantation or for whom a matching organ cannot be found, the VAD is an essential therapeutic option as destination therapy. The number of VAD implantations during the last 5 years has increased exponentially. In patients with left ventricular assist device (LVAD) ischemic stroke and intracranial hemorrhage are major causes of morbidity (38). According to the annual reports of INTERMACS 2015 (39) and 2017 (40) bleeding was the most frequent complication in patients with VAD-support. The development of improved and smaller VADs which produce less shear stress and cause less complications is elementary.

## Acquired von Willebrand Syndrome in Patients With VAD Support

Geisen et al. were the first to showed that CF-VAD patients develop Acquired von Willebrand syndrome (AVWS) (41). The adhesive character of the surface within the VADs and the altered flow conditions in CF-VAD patients cause an increased interaction of blood components with the VAD surfaces which initiates increased platelet adhesion and activation leading to an enhanced activation of coagulation (42, 43). This can cause thromboembolic episodes and therefore, adequate anti-thrombotic therapy is needed.

AVWS develops due to increased shear stress caused by the VAD. Characteristically for AVWS is the loss of the HMW-multimers which leads to reduced VWF collagen binding activity (VWF:CB), ristocetin cofactor activity (VWF:RCo), and VWF:Activity (VWF:Ac), respectively. This translates in a reduction of the corresponding VWF:CB/VWF-Antigen (VWF:CB/VWF:Ag)-ratio, VWF:RCo/VWF-Antigen (VWF:RCo/VWF:Ag)-ratio, and VWF:Act/VWF-Antigen (VWF:Ac/VWF:Ag)-ratios, respectively (44–46).

Geisen et al. (41) investigated VWF parameters in patients with CF-VAD and in patients after HT. As acute phase proteins VWF:Ag and C -reactive protein were increased in both cohorts, however, VWF:CB and VWF:RCo-factor, respectively, were decreased only in VAD-patients leading to reduced VWF:CB/VWF:Ag and VWF:RCo/VWF:Ag ratios respectively. In addition, all the CF-VAD patients presented a loss of the VWF HMW-multimers, i.e., these patients had AVWS. In patients with HT, both the VWF:CB/VWF:Ag and VWF:RCo/VWF:Ag ratios and the VWF multimeric analyses were normal (41).

Heilmann et al. described that most of the CF-VAD patients developed AVWS within 1 day (46). Further investigations revealed that the acquired VWF defect is already present within 3 h after VAD-implantation (30) and is totally reversed after CF-VAD weaning (10, 38).

## Clinical Implications of AVWS in VAD-patients

Bleeding is the most common adverse event after implantation of continuous flow VADs. As observed in other causes of AVWS (47), perioperative and mucosal bleeding account for most events In VAD-patients (48). GI bleeding is a hallmark of continuous flow VAD support, with rates up to 10-fold higher with early experiences of continuous flow LVADs than with the previous pulsatile devices (49). Genovese et al. reported that almost 50% of the investigated VAD patients suffered from bleeding symptoms within the first 60 days after VAD-implantation and almost 25% of the these patients needed surgical intervention for bleeding complications (50). Klovaite et al. observed that 75% of their LVAD-patients suffered from bleeding complications (51). GI-bleeding occurs more often from digestive angiodysplasia (10) and is characterized by a high recurrence rate up to 40% in a recent multi-center study (52).

Heilmann et al. investigated 74 patients with HeartMate-2 for 6.5 years and demonstrated that AVWS is a persisting problem in these patients (53). Geisen et al. showed that the VWF:Activity-assay (VWF:Ac, Innovance VWF Ac) is better than the VWF:RCo-assay to recognize AVWS (54). Further analyses revealed that our *in-house* VWF:CB-assay is the more sensitive assay for the detection of VWF HMW-multimers because in this assay collagen type I is used which has a higher affinity for VWF HMW multimers than collagen type III, and is therefore more discriminative for AVWS (55). In the largest CF-VAD cohort investigated for VWF defect, AVWS was detected in all patients whatever the nature of the device or their combination (56). In this cohort, VWF:CB/VWF:Ag ratio was most often reduced in patients with biventricular Assist Device (BVAD, consisting of two Thoratec paracorporal VAD) and in patients with HeartMate-2 in combination with a right ventricular assist device. These patients presented also most often with bleeding symptoms. Interestingly, in patients with HeartMate-3 the reduction of VWF:CB/VWF:Ag-ratio and the loss of the HMW-multimers were less severe compared to others VAD-patients.

Interestingly, Heilmann et al. demonstrated for the first time that patients with venoarterial ECMO also develop AVWS and that these patients present with increased bleeding symptoms (28). ECMO pumps also provide enhanced shear stress (57) inducing platelet activation and dysfunction. In this situation, the association of AVWS and platelet dysfunction can lead to increased bleeding risk including life-threatening complications. When performing platelet aggregometry in VAD-patients, Baghai et al. demonstrated an impaired platelet function that was not restricted to reduced ristocetin-induced aggregation (secondary to the HMW-multimers defect). Further flow cytometry analysis revealed that VAD-patients also suffered from a platelet α- and δ-granules secretion defect which may further enhance the bleeding tendency (58). Other investigations in patients supported with venovenous ECMO showed that all of them rapidly developed AVWS, thrombocytopenia and platelet dysfunction, resulting in severe impairment of coagulation. Aggregometry revealed impaired platelet function after agonist stimulation by adenosine diphosphate, ristocetin, collagen, and epinephrine. Flow-cytometric platelet analyses demonstrated severely decreased CD62- and CD63-expression on platelets. After explantation, VWF parameters normalized within hours (59). CF-MCS might also impair platelet function through promoting the shedding of platelet receptors GPIbα and GPVI (60).

Sakatsume et al. reported that patients with continuous flow LVAD support and with GI-bleeding exhibited a more severe loss of VWF HMW-multimers compared to patients without GI-bleeding (61). However, Meyer et al. didn't find such association between the intensity of HMW-multimers defect and the rate of bleeding complications in a series of 102 patients (27). Whether monitoring VWF, platelet count, and platelet function could help to stratify bleeding risk and optimize anticoagulation therapy and outcomes in CF-VAD patients needs further research.

## Pathophysiology of Bleeding Related to AVWS in VAD-patients

Despite VWF HMW-multimers defect is near universal with VAD therapy, not all patients have bleeding events, indicating a multifactorial process. Moreover, bleeding is mainly related to the development of arteriovenous malformations (AVM) in gastrointestinal and nasal tracts. Whether these AVM are pre-existing or result from *de novo* angiogenesis from the gut and nasal mucosa is unknown. According to the “2-hit” model, several hemodynamic, anatomic and hematologic abnormalities could drive the development of symptomatic AVM in VAD-patients. Bleeding complications could also be favored by different injuries in mucocutaneous areas including septic challenges. It is also proposed that gut hypoxia resulting from reduced cardiac output in heart failure patients and lowered pulse pressure induced by LVAD therapy could result in upregulation of angiogenic mediators and lead to the development of mucosal angiodysplasia that would become symptomatic in the context of AVWS and anticoagulation (62). Alternatively, Tabit et al. have recently proposed that increased thrombin generation generated by blood contact pathway activation at the interface of VAD foreign surface could be a main trigger of angiogenesis. In this study, plasma sampled from LVAD patients had higher serum levels of Ang-2 and promoted endothelial angiogenesis *in vitro* through thrombin-induced Ang-2 expression (63).

Several studies raised also interest about a possible causal relationship between acquired VWF defect and the development of AVM in VAD-patients. This hypothesis comes both from long-standing clinical observations in constitutional VWD and recent experimental studies suggesting that VWF regulates angiogenesis through multiple pathways. GI-bleeding from angiodysplasia is frequently observed in constitutional VWD but almost exclusively in patients lacking VWF HMW-multimers (64). It is now established from *in vitro* and *in vivo* studies that lack of VWF causes enhanced vascularization, both constitutively and following ischemia. The molecular pathways are likely to involve VWF binding partners (such as integrin αvβ3), WBPs components (such as Ang-2 and galectin-3 whose storage is regulated by VWF) and to converge to vascular endothelial growth factor signaling, a key regulator of angiogenesis(65, 66).

CF-VAD was recently reported to contribute by itself to the development of small-bowel AVM through increased angiogenesis in a dedicated animal model. Moreover, it was inferred from *in vitro* culture assays that VWF degradation products resulting from HMW-multimers cleavage could have a direct pro-angiogenic effect on gut microvessels (67). According to this interesting hypothesis, the imbalance between VWF high and low molecular weight multimers rather than the lack of HMW-multimers would account for the GI-bleeding events in VAD-patients. However, it does not explain why GI-bleeding from AVM is also frequently observed in VWD-type 3.

## Therapeutic Options for Bleeding Management of VAD-patients: Pros, Cons, and Challenges

As in non-VAD-patients, endoscopy remains the standard of care to manage GI-bleeding in VAD-patients. If conventional upper and lower endoscopies fail to identify a bleeding lesion, a wireless videocapsule endoscopy should be performed to localize the source of bleeding, to risk stratify the patient and to guide further endoscopic interventions. Indeed, some angiodysplasia might be eligible to endoscopic treatment with balloon enteroscopy using argon plasma coagulation or endoclips.

Because different types of VADs cause different ranges of shear stress and complications such as thromboembolism or bleeding, the antithrombotic regimens vary considerably nationally and internationally. Heparin, vitamin K antagonists (VKA) and antiplatelet agents are applied partly depending on the use of the different devices. Unfractionated heparin bridging is recommended after LVAD implantation to bridge to therapeutic VKA anticoagulation. VKA therapy is the only long-term anticoagulant approved for LVAD patients. A randomized pilot study showed an unacceptable rate of thromboembolic complications in LVAD-patients treated with dabigatran compared to those treated with VKA (68). A reduced anticoagulation strategy following a bleeding event is also associated to a higher risk of device thrombosis and ischemic stroke (69).

In patients with refractory or recurrent GI-bleeding or in whom a lesion cannot be localized or treated by endoscopy, others approaches might be discussed according to the “2-hit model: these aiming to prevent or eradicate angiodysplasias and/or those aiming to reduce or prevent HMW-multimers defect. Data supporting the use of antiangiogenic drugs, mostly octreotide (52, 70–73) and thalidomide (74–76), are sparse and mostly limited to case reports and retrospective single-center studies. In constitutional VWD, recurrent GI-bleeding is an indication to start prophylaxis with intravenous infusion of VWF concentrates. With only one case report published (77), there is currently no evidence supporting the efficacy and safety of such approach in VAD-patients. In this specific setting, VWF-containing concentrates may have limited efficacy due to the short half-life of VWF (30). Some efficacy could be observed in single cases (unpublished experience) (Table 1).

**Table 1:** Published Literature on the management of GI-bleeding in LVAD patients with antiangiogenic drugs or VWF concentrate.

**Authors**	**Patients treated**	**Indication**	**Medication**	**Outcome**	**Adverse events**
Loyaga-Rendon et al. (70)	7	Secondary prophylaxis of GI-bleeding	Octreotide	No significant reduction in hospitalizations, transfusion need, or number of endoscopies at 3 months	Abdominal pain Diarrhea
Aggarwal et al. (71)	10	Secondary prophylaxis of GI-bleeding	Secondary prophylaxis	No difference in length of hospitalization, GIB recurrence rate, or transfusion need	None reported
Hayes et al. (72)	5	Secondary prophylaxis of GI-bleeding	Octreotide	Successfully treated	None reported
Malhotra et al. (73)	10	Primary prophylaxis of GI-bleeding	Octreotide	No GI-Bleeding events	None reported
Shah et al. (52)	51	Secondary prophylaxis of GI-bleeding	Octreotide	Lower recurrence of GI bleed compared to a matched historical control group (24 vs. 43%; *p* = 0.04)	None reported
Draper et al. (74)	8	Secondary prophylaxis of GI-bleeding	Thalidomide	5 patients had no recurrence of bleeding 2 patients had reduction of bleeding 1 patient died within 1 week of initiation	Neuropathy, Sepsis
Ray et al. (75)	1	Secondary prophylaxis of GI-bleeding	Thalidomide	No recurrent bleeding at 1 year	No thrombosis at 1 year
Seng et al. (76)	11	Secondary prophylaxis of GI-bleeding	Thalidomide	Recurrent GIB occurred in 4 patients (45.4%) post-discontinuation of thalidomide therapy	1 pump thrombosis 1 Neuropathy
Fischer et al. (77)	1	Refractory GI-bleeding	VWF concentrate 80 IU/kg daily	1st GI-bleeding event successfully treated. Restart of VWF therapy with octreotide after recurrence of GI-bleeding	No thrombosis

In these challenging patients, the development of targeted drugs preventing HMW-multimers degradation without increasing platelet adhesion and aggregation could be an attractive option. Doxycycline was reported to inhibit proteolytic cleavage of VWF in the presence of LVAD-associated shear stress but at plasma concentrations 10-fold higher than the levels obtained with standard dosing (78). A monoclonal antibody anti-VWF D4 domain partially inhibiting VWF binding to ADAMTS13 was also reported to prevent endogenous HMW-multimers cleavage in a MCS mock circulatory loop model (32).

## Conclusions

In AVWS developed in all patients with CF-VAD implantation and seems to convey a higher risk of GI-bleeding from angiodysplasia. Smaller devices with less complication rates are needed. During upcoming years the outcome (especially regarding complication rates) novel devices (for example HeartMate 3 or HeartWare MVAD) will be further investigated. Another often discussed question is the optimal timeframe of VAD initiation to provide the best clinical outcome. Since the development angiodysplasias under CF-VAD remains poorly understood, this research field needs to be further investigated.

## Author Contributions

AR, SS, and BZ wrote sections of the manuscript. All authors contributed to manuscript revision, read and approved the submitted version.

### Conflict of Interest Statement

The authors declare that the research was conducted in the absence of any commercial or financial relationships that could be construed as a potential conflict of interest.
